# Effect of wild yeast inoculation on the nutritive value, fermentation profile, and aerobic stability of rehydrated corn grain silage

**DOI:** 10.3168/jdsc.2025-0888

**Published:** 2025-12-04

**Authors:** M.R. Pupo, E.C. Diepersloot, M.P.R. Wenzel, L.F. Ferraretto

**Affiliations:** Department of Animal and Dairy Sciences, University of Wisconsin–Madison, Madison, WI 53706

## Abstract

Feeding spoiled silage with high yeast activity is commonly related to impaired lactation performance by dairy cows. However, rehydrated corn grain silage (RCGS) may be more prone to yeast proliferation and be greatly affected by high yeast populations. This experiment assessed the effects of increasing wild yeast counts on the nutrient composition, fermentation profile, and aerobic stability of RCGS at different storage lengths. Wild yeast was obtained after exposing high-moisture corn to an aerobic environment for 10 d. The experiment consisted of 4 treatments (theoretical concentration of 0 [only distilled water; CON], 5.3 [low yeast; LY], 6.3 [medium yeast; MY], and 7.3 [high yeast; HY] log cfu/g of RCGS) for either 30 or 90 d of storage in quadruplicate (used as blocking factor), for a total of 32 laboratory silos. Minimal changes in nutrient composition and aerobic stability were detected regardless of treatment and storage length. At 30 d, acetic acid and ethanol concentrations showed a positive linear relationship with increasing wild yeast count. Greater yeast count was observed in silage inoculated with wild yeast compared with CON, but no differences were detected among inoculated silage. The 7-h in vitro DM degradability linearly decreased with increasing wild yeast counts. At 90 d, total acid concentration was greater for HY compared with other treatments, with a positive quadratic relationship when increasing wild yeast count. Similarly, lactic acid concentration was greater for HY compared with other treatments, with a positive linear relationship when increasing wild yeast count. In addition, HY had greater ethanol concentration compared with other treatments, with a positive linear relationship when increasing wild yeast count. The 7-h in vitro DM degradability was quadratically related to wild yeast population. Overall, this study provides additional information regarding the nutrient composition, fermentation profile, and aerobic stability of RCGS with wild yeast inoculation. Moreover, high wild yeast populations can reduce in vitro DM degradability, which could potentially affect performance of dairy cows. Further research is warranted to identify wild yeast populations as their presence is dependent on their species and metabolism.

## Highlights

• Wild yeast inoculation did not largely influence silage nutritive value.
• Silage had greater yeast counts and a less pronounced fermentation.
• Ethanol concentration linearly increased with increasing wild yeast inoculation.
• High wild yeast populations reduced 7-hour in vitro DM degradability.

## Graphical Abstract

**Figure:**
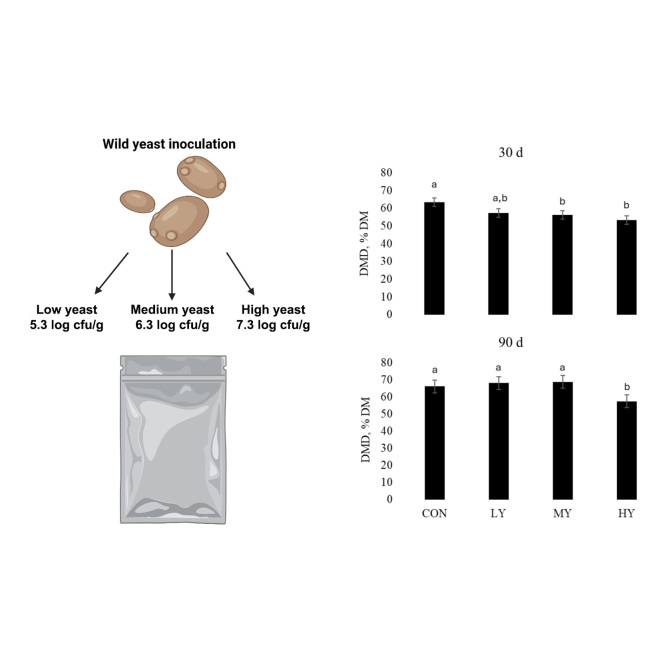
**Summary:** In the current study, wild yeast was obtained from high-moisture corn that was exposed to an aerobic environment for 10 days to promote yeast growth. Dry ground corn was rehydrated targeting 70% dry matter (DM) and inoculated with wild yeast to achieve final yeast concentrations of 0, 5.3, 6.3, and 7.3 log colony forming units/mL. Silos were placed in vacuum pouches and stored for 30 or 90 days. Increasing wild yeast inoculation did not largely influence silage nutritive value. However, high wild yeast population reduced 7-hour in vitro DM degradability, which in turn could potentially affect performance of dairy cows.

Yeast counts play a key role in determining the fate of silage when silos are opened (Santos et al., 2017) and yeast counts at opening are influenced by the number of yeast present at ensiling and how well they survive during fermentation. When silage is exposed to oxygen (e.g., open silos, leaky silos, holes in bag silos, poorly packed silage), lactate-assimilating yeasts are often the primary initiators of aerobic spoilage (McDonald et al., 1991). These microorganisms are active at low pH and can degrade lactic acid, thereby increasing silage pH to an extent that allows opportunistic bacteria and molds to grow (Kung et al., 2018). The main challenges associated with the presence of yeasts in silage are associated with greater DM losses (Carvalho et al., 2014), undesirable fermentation (particularly clostridial or butyric acid-based), and lower aerobic stability of silage (Weiss et al., 2016). Furthermore, starch concentration is high in corn grain silage, which can be used as a source of energy for yeasts (Kung et al., 2018). During the fermentation process, yeasts can ferment starch to ethanol, but when oxygen is present, these microorganisms can trigger spoilage that reduces silage nutritive value (McDonald et al., 1991). Because corn grain silage can have approximately 40 percentage units greater starch concentration compared with whole-plant corn silage, it may be more prone to yeast proliferation and be affected to a greater magnitude when high yeast populations are present (Kung, 2010). Interestingly, few studies have assessed the effects of feeding spoiled silage to dairy cows. According to Santos et al. (2015), spoiled silage can be divided into those actively spoiling (yeast counts ≥5.5 log cfu/g, temperature ≥36°C, and low concentrations of organic acids) and those that have spoiled and been stored for prolonged periods. Hoffman and Ocker (1997) observed that mid-lactation cows fed a TMR containing spoiled high-moisture corn (**HMC**) silage had 3 kg/d lower milk yield compared with cows fed control silage. Likewise, Whitlock et al. (2000) reported that feeding actively spoiling silage decreased DMI of steers compared with stable silage. Because spoiled silage typically contains high yeast populations, there is growing interest in their potential negative effects on animal performance. Although a previous study (Santos et al., 2015) isolated a single yeast colony and added to in vitro culture tubes containing TMR to achieve final yeast concentrations of 0, 4.40, 6.40, and 8.40 log cfu/mL, research evaluating the effects of wild yeasts inoculated directly into silage are still lacking. It is well known that spoilage is triggered by different yeast populations rather than specific strains (Ohyama et al., 1975). Beck and Gross (1964) observed that silage deterioration was related to yeast counts. Likewise, Kung et al. (1998) observed a negative relationship between yeast counts and aerobic stability, especially in corn-based crops.

Thus, the objective of this experiment was to assess the effects of increasing wild yeast counts on the nutrient composition, fermentation profile, and aerobic stability of rehydrated corn grain silage (**RCGS**) at different storage lengths. Our hypothesis was that increasing wild yeast counts would negatively affect nutritive value, fermentation profile, and aerobic stability of RCGS.

This experiment was carried out in a completely randomized block design with 4 treatments (wild yeast inoculations) for either 30 or 90 d of storage in quadruplicate (used as blocking factor) for a total of 32 laboratory silos.

To obtain the wild yeast that was later used to inoculate RCGS in this experiment, HMC was collected from the University of Wisconsin–Madison Arlington Agricultural Research Station (Arlington, WI) on December 2, 2023, using a combine (Axial-Flow 6150, Case IH, Racine, WI) and ground using a hammer mill (RH-A 2248, Renn Mill Center Inc., Lacombe, AB, Canada; 746 µm geometric mean particle size). Then, HMC was exposed to an aerobic environment for 10 d to promote yeast growth. Briefly, HMC was collected and around 8 kg of feed was placed into a 20-L plastic bucket (Uline, Kenosha, WI), covered with cheesecloth, and maintained at a temperature of approximately 40°C for the entire period of aerobic exposure (Zakhartsev et al., 2015). After 10 d, a representative sample of HMC was collected and subsequently, 20 g of undried, unground subsample was diluted 10-fold (mass basis) in 0.1% peptone water (Dot Scientific Inc., Burton, MI), blended for 60 s in a high-speed blender, and filtered through 2 layers of cheesecloth to collect the silage extract. The extract was used to grow yeast via a pour plating method in a 10-fold serial dilution on malt extract agar (Difco 211220) treated with 0.1% chloramphenicol. Agar plates were incubated at 32°C for 48 h, after which 10 to 15 individual yeast colonies (identified by visual examination of colonies) were collected and composited together in growth medium (YPD broth, Dot Scientific Inc., Burton, MI) treated with 0.1% chloramphenicol. The yeast and growth media were incubated in sealed flasks at 32°C for 72 h; then all flasks were combined and mixed to inoculate RCGS. To enumerate viable cells for inoculation into RCGS, 1 mL of a 100 × dilution of the composited growth medium was mixed into 0.1 mL of trypan blue stock solution, resulting in a final concentration of 0.04% wt/vol. Then, 10 µL of this solution was pipetted into a Neubauer chamber (Gebeauty Ree, Miami, FL) and the cells were counted using an optical microscope. The growth media had a final wild yeast concentration of 9.3 log cfu/mL.

Four samples of dry ground corn (94% DM; 616 ± 78 µm geometric mean particle size) were obtained from the University of Wisconsin–Madison Arlington Agricultural Research Station Feed Mill and used as 4 replicates (used as blocking factor) to prepare RCGS. Each sample was from an independent load that arrived at the mill and was collected 1 to 2 wk apart. Each of these samples was homogenized and split into 8 subsamples, which were randomly allocated to each treatment by storage length combination. Yeast and growth media were diluted with enough distilled water to achieve a theoretical concentration of 0 (only distilled water; **CON**), 5.3 (low yeast; **LY**), 6.3 (medium yeast; **MY**), and 7.3 (high yeast; **HY**) log cfu/g of RCGS. Typically, spoiled corn-based silage samples contain around 7 log cfu/g of wet silage (Ward, 2011). The diluted growth medium (or distilled water) was combined with dry ground corn targeting 70% DM for each replicate and mixed. Subsequently, 1,500 g of RCGS was placed in nylon-polyethylene standard barrier vacuum pouches (0.09 mm thickness, 25.4 × 35.6 cm; Doug Care Equipment Inc., Springville, CA) and vacuum-sealed using an external clamp vacuum machine (Bestvac; distributed by Doug Care Equipment). Silos were stored for 30 or 90 d. All silos were stored at room temperature until they reached their assigned storage length. After each specified storage length was reached, the remaining material from each individual laboratory silo was mixed, and representative subsamples were collected for analysis. After subsamples were collected, silage was evaluated for aerobic stability using the remaining material.

Extracts of silage after each storage length period were obtained from the homogenization of an undried and unground sample as described previously. The extract was divided into 2 subsamples. The first subsample was used to determine pH. Measurements of pH were conducted by using a pH meter (Thermo-Orion Dual Star, Thermo Fisher Scientific Inc.) fitted with a glass pH electrode (Thermo-Orion 9172BNWP, Thermo Fisher Scientific Inc.). In addition, the second subsample of the extract was added to a 50-mL plastic tube to evaluate yeast and mold enumeration via a pour plating method in a 10-fold serial dilution on malt agar (Difco 211220) acidified with 85% lactic acid. Agar plates were incubated at 32°C for 48 h for yeast, and an additional 72 h for mold counts. Yeast and mold counts were evaluated on the same plates and separated by visually examining the growth of colonies.

Subsamples reserved for analysis of silage fermentation products were sent to a commercial laboratory (Dairyland Laboratories Inc., Arcadia, WI) for quantification of ammonia, organic acids (lactic, acetic, propionic, valeric, butyric, and iso-butyric acid), ethanol, and 1,2-propanediol. Silage fluid was extracted by blending 15 g of silage into 200 mL of distilled water for 2 min. The filtrate was centrifuged at 4°C and 21,500 × *g* for 20 min; lactic, acetic, propionic, valeric, butyric, and iso-butyric acids, 1,2-propanediol, and ethanol were measured in the supernatant fluid using the method of Canale et al. (1984) and a high-performance liquid chromatograph (L7485, Hitachi, Tokyo, Japan) fitted with an UV detector (Spectroflow 757, ABI Analytical Kratos Division, Ramsey, NJ). Ammonia concentration was evaluated by colorimetry using a Technicon Automatic Analyzer (RFA-300, Alpkem Corporation, Wilsonville, OR) with a method adapted from Noel and Hambleton (1976).

Representative fermented samples from each laboratory silo at opening were dried in a forced-air oven set at 60°C for 48 h to determine DM concentration, and ground to pass through a 1-mm sieve using a Wiley Mill (Thomas Scientific, Swedesboro, NJ) for chemical characterization. Dried and ground samples were analyzed for ether extract, CP, starch, and NDF using near-infrared spectroscopy (**NIRS**) with a Foss 2500 (Foss-NIR System, Silver Springs, MD). Calibration equations for NIRS analysis of ether extract, CP, starch, and NDF were based on wet chemistry procedures described in method 920.39 (AOAC International, 2016), method 990.03 (AOAC International, 2016), Vidal et al. (2009), and method 2002.04 (AOAC International, 2016), respectively. The SE of cross-validation for ether extract, CP, starch, and NDF determined using α-amylase and sodium sulfite (**aNDF**) NIRS calibrations were 0.40, 0.48, 2.57, and 1.66, respectively.

The 7-h in vitro DM degradability (used as a proxy of starch degradability) of RCGS was determined using the in vitro procedure described by Goeser and Combs (2009). Triplicate samples (0.5 ± 0.001 g) were weighed into F57 bags (Ankom Technologies, Macedon, NY) and incubated within 125-mL wide-neck glass bottles, containing rumen fluid, buffer medium, plus micro- and macrominerals according to Goering and Van Soest (1970). These flasks were purged continuously with CO_2_ and maintained in a water bath at 39°C during incubation period. Briefly, rumen fluid was collected from 2 multiparous, ruminally cannulated Holstein cows housed at the University of Wisconsin–Madison Dairy Cattle Center (Madison, WI) fed a diet consisting of 27% DM corn silage, 31% DM alfalfa silage, 36% DM concentrate mix, and 6% DM whole cottonseed. The animal research portion of this study was conducted under an approved protocol by the Animal Care and Use Committee of the College of Agriculture and Life Sciences at the University of Wisconsin-Madison.

Laboratory silos containing approximately 1 kg of remaining silage were placed into individual 4-L plastic buckets (Uline, Kenosha, WI) to evaluate aerobic stability. Two temperature sensors (HOBO temperature data logger 64 k, Onset Computer Corp., Cape Cod, MA) were placed in the geometric center of the bucket and temperature was recorded every 30 min. Three additional sensors were used to monitor room temperature in the event of temperature fluctuations. Room temperature averaged 23.1 ± 1.01°C among all periods of aerobic stability measurements. Buckets were covered with 2 layers of cheesecloth to prevent drying, and silos were left exposed to aerobic conditions for 240 h. Aerobic stability was defined as the number of hours until silo temperature increased 2°C above the baseline silo temperature.

Samples from 30 and 90 d of storage were analyzed separately to evaluate the effects of yeast inoculation. Laboratory silos were experimental units. Normality of residuals was assessed with the Shapiro–Wilk test using the univariate procedure in SAS (version 9.4, SAS Institute Inc., Cary, NC). Because microbial data did not meet assumptions of normality, data were log_10_-transformed before statistical analysis and presented as log_10_ values. For each storage length, data were analyzed as a completely randomized block design using a general linear mixed-model procedures (PROC GLIMMIX, SAS 9.4; SAS Institute Inc.) with a model including the fixed effect of wild yeast inoculation. Replicate (used as blocking factor) was the sole random effect. Means were determined using the LSMEANS statement and treatment means were compared using the DIFF LINES option. Orthogonal contrasts were used to evaluate linear and quadratic effects of wild yeast inoculation (from 0 to 7.3 log cfu/g of RCGS). Because treatments were unequally spaced, contrast coefficients were determined using PROC IML of SAS. Significance was declared at *P* ≤ 0.05.

Propionic acid, 1,2-propanediol, valeric acid, and iso-butyric acid were measured, but not detected in any treatments. Mold counts were also evaluated but below detection limit (2.0 log cfu/g of RCGS) for all samples. Minimal effects of wild yeast inoculation were observed for DM (71.5% ± 0.83% as fed), CP (7.85% ± 0.42% DM), NDF (10.2% ± 0.43% DM), starch (78.4% ± 0.53% DM), soluble CP (44.9% ± 0.64% CP), ether extract (4.33% ± 0.47% DM), and ash (0.96% ± 0.16% DM) concentrations in the current study (data not presented in tables). The effect of wild yeast inoculation on the pH, fermentation profile, and microbial counts of RCGS stored for 30 d are presented in Table 1. A positive quadratic relationship (*P* = 0.03) with increasing wild yeast inoculation was observed for pH. Although lactic acid is typically the main source of energy for yeast in the first phase of silage deterioration (Kung et al., 2018), no changes (*P* = 0.75) were observed for lactic acid concentration that could explain increased silage pH. In addition, acetic acid linearly increased (*P* = 0.001) with increasing wild yeast count. Perhaps, an increase in silage pH in this study could have been triggered by other undesirable microorganisms, such as acetic acid bacteria or enterobacteria. In corn-based silage, acetic acid-producing bacteria can participate in aerobic deterioration (Woolford, 1990). Spoelstra et al. (1988) suggested that aerobic deterioration is typically initiated by a concomitant increase in yeast and acetic acid-producing bacteria populations. These bacteria oxidize residual sugars and organic acids, thereby increasing pH. Expectedly, ethanol concentration linearly increased (*P* = 0.001) with increasing wild yeast count. Nordström (1966) found that greater concentration of ethanol in silage was associated with number of yeasts and related to certain species that can form esters. Similarly, Kung et al. (2018) reported that increases in ethanol concentration are frequently related to high numbers of yeasts, and such silages are likely to spoil when exposed to air. Greater yeast count was detected (*P* = 0.001) with wild yeast inoculation (LY, MY, and HY) but did not differ among them. Kung et al. (2018) previously described that numbers of yeasts on the fresh samples of whole-plant corn forage were not correlated with the final number of yeasts in the resulting silage. Likewise, Weinberg et al. (2011) reported that high initial yeast numbers can remain the same, be inhibited, or increase during storage. The factors affecting the activity of yeasts are complex interactions between the significant diversity of microorganisms, causing variations in silage (Weiss et al., 2016). For example, Duniere et al. (2015) reported that substrate competition can affect yeast population, both in number and composition, in silage. Therefore, final yeast counts in silage may not be determined solely by initial yeast numbers in the pre-ensiling material. Further research is needed to elucidate processes involved in yeast population dynamics during ensiling. Surprisingly, mold counts were below detection limits for all samples, regardless of storage length. Molds are generally less acid-tolerant than yeasts, so their growth is normally observed after yeast growth (Borreani et al., 2014). One possible explanation is that RCGS had restricted fermentation due to the lack of substrate or low moisture concentration (or both), which may have limited the growth of these microorganisms (Kung et al., 2018). Furthermore, the lack of effect (*P* = 0.23) on RCGS aerobic stability with inoculated wild yeast can be attributed to low aerobic stability of corn grain silage, regardless of addition of wild yeast. Stevenson (1976) reported that most ensiled corn grain begins to deteriorate in less than 24 h, which agrees with our findings. Moreover, it is likely that the low moisture concentration of RCGS restricted the activity of inoculated wild yeast (Goodrich and Meiske, 1976), which in turn influenced the lack of effect on aerobic stability.

**Table 1:** The effect of wild yeast inoculation on the pH, fermentation profile, and microbial counts of rehydrated corn grain silage (RCGS) within 30 d^1^

Item	CON	LY	MY	HY	SEM^3^	*P*-value^2^	
						L	Q
pH	4.55^b^	4.85^a^	4.81^a^	4.75^ab^	0.10	0.10	0.03
Total acids, % DM	0.99	1.00	1.20	1.23	0.20	0.19	0.95
Lactic acid, % DM	0.83	0.76	0.91	0.93	0.17	0.44	0.73
Acetic acid, % DM	0.12^b^	0.21^a^	0.24^a^	0.27^a^	0.03	0.001	0.18
Butyric acid, % DM	0.05	0.03	0.05	0.03	0.01	0.14	0.86
Ethanol, % DM	0.57^c^	1.33^b^	1.69^a^	1.90^a^	0.12	0.001	0.01
Ammonia-N, % CP	2.53	2.58	2.81	2.63	0.28	0.57	0.57
Yeast, log cfu/g	3.36^b^	5.47^a^	5.73^a^	5.68^a^	0.18	0.001	0.001
Aerobic stability, h	12.1	17.5	15.6	13.4	3.26	0.86	0.13

a–c Means within a row with different superscripts denote an effect of wild yeast inoculation within 30 d (*P* ≤ 0.05).

1 CON = no addition of wild yeast; LY = addition of wild yeast to achieve projected counts of 5.3 log cfu/g of RCGS; MY = addition of wild yeast to achieve projected counts of 6.3 log cfu/g of RCGS; HY = addition of wild yeast to achieve projected counts of 7.3 log cfu/g of RCGS.

2 L = linear effect and Q = quadratic effect of wild yeast inoculation.

3 Greatest SEM.

The effects of wild yeast inoculation on the pH, fermentation profile, and microbial counts of RCGS stored for 90 d are presented in Table 2. Although pH was not affected by wild yeast inoculation (*P* = 0.43) at 90 d of storage, the pH of RCGS declined to within the normal range of pH for HMC (Kung et al., 2018). Total acid concentration was greater (*P* = 0.002) for HY compared with other treatments, with a positive quadratic relationship (*P* = 0.02) when increasing wild yeast count. Similarly, lactic acid concentration linearly increased (*P* = 0.001) with increasing wild yeast count. Kung et al. (2007) previously described that low accumulation of total acids would be expected for silage with reduced water-soluble carbohydrate concentration, low moisture concentration, or both. Therefore, the reason behind these findings in the current study is unclear. Similar to 30 d of storage, ethanol concentration linearly increased (*P* = 0.001) with increasing wild yeast count. Even under anaerobic silage conditions, undesirable microorganisms are capable of fermenting sugars to ethanol and CO_2_ (McDonald et al., 1991). Despite the lack of effect (*P* = 0.97) on RCGS aerobic stability with inoculated wild yeast at 90 d, increased aerobic stability compared with RCGS stored for 30 d was likely associated with gradual accumulation of fermentation products with antifungal properties, such as acetic acid. Likewise, da Silva et al. (2019) observed that aerobic stability of RCGS increased from 50 h at 30 d to approximately 150 h at 90 d, similar to our findings.

**Table 2:** The effect of wild yeast inoculation on the pH, fermentation profile, and microbial counts of rehydrated corn grain silage (RCGS) within 90 d^1^

Item	CON	LY	MY	HY	SEM^3^	*P*-value^2^	
						L	Q
pH	4.25	4.35	4.30	4.25	0.07	0.89	0.14
Total acids, % DM	1.60^bc^	1.51^c^	1.84^b^	2.30^a^	0.14	0.001	0.02
Lactic acid, % DM	1.15^b^	1.22^b^	1.45^b^	1.85^a^	0.15	0.001	0.15
Acetic acid, % DM	0.33	0.25	0.31	0.42	0.07	0.18	0.08
Butyric acid, % DM	0.13^a^	0.04^b^	0.08^ab^	0.04^b^	0.03	0.04	0.36
Ethanol, % DM	0.65^c^	1.29^b^	1.53^b^	1.95^a^	0.11	0.001	0.19
Ammonia-N, % CP	3.58	3.53	3.67	3.77	0.17	0.21	0.56
Yeast, log cfu/g	2.72^b^	3.25^ab^	4.55^a^	3.19^b^	0.42	0.08	0.03
Aerobic stability, h	205.3	184.8	186.6	240.0	41.3	0.86	0.13

a–c Means within a row with different superscripts denote an effect of wild yeast inoculation within 90 d (*P* ≤ 0.05).

1 CON = no addition of wild yeast; LY = addition of wild yeast to achieve projected counts of 5.3 log cfu/g of RCGS; MY = addition of wild yeast to achieve projected counts of 6.3 log cfu/g of RCGS; HY = addition of wild yeast to achieve projected counts of 7.3 log cfu/g of RCGS.

2 L = linear effect and Q = quadratic effect of wild yeast inoculation.

3 Greatest SEM.

The effect of wild yeast inoculation on the 7-h in vitro degradability of DM from RCGS stored for 30 and 90 d and incubated with varying amounts of wild yeasts are presented in Figure 1. The DM degradability is a suitable parameter to estimate starch degradability in corn grain silage, mainly because starch is the major component of corn kernels (Zinn et al., 2002). At 30 d, DM degradability linearly decreased (*P* = 0.004) with increasing wild yeast count. At 90 d, a quadratic effect was observed (*P* = 0.02) on DM degradability. Santos et al. (2015) isolated a spoilage yeast (*Issatchenkia orientalis*) from HMC and inoculated ruminal fluid causing a decrease in vitro fiber digestibility from a TMR incubated for 12 and 24 h. Similarly, Whitlock et al. (2000) reported up to 8.5 percentage units lower in vivo DM digestibility for steers fed normal versus spoiled whole-plant corn silage. The same authors also observed that feeding spoiled silage caused partial or total destruction of the ruminal mat phase. These results indicate that dairy cows fed silage with high wild yeast populations (≥7.3 log cfu/g of forage) may have impaired ruminal nutrient digestibility. Although ethanol concentration was greater for HY compared with other treatments, these differences only partially explain the changes in DM degradability observed among treatments in this study. One possible explanation is that spoiled silage could also comprise mycotoxin-producing molds and other undesirable microorganisms (i.e., *Listeria monocytogenes* and *Clostridium botulinum*), which in turn can affect animal health and performance (McDonald et al., 1991). For example, Salvo et al. (2015) fed corn silage inoculated with *Pichia norvegensis* and reported that component-corrected milk yield decreased by 1.3 kg/d without affecting DMI of dairy cows. Our findings suggest negative effects on silage nutrient availability in addition to the effects reported by previous literature on ruminal fermentation.

**Figure 1:**
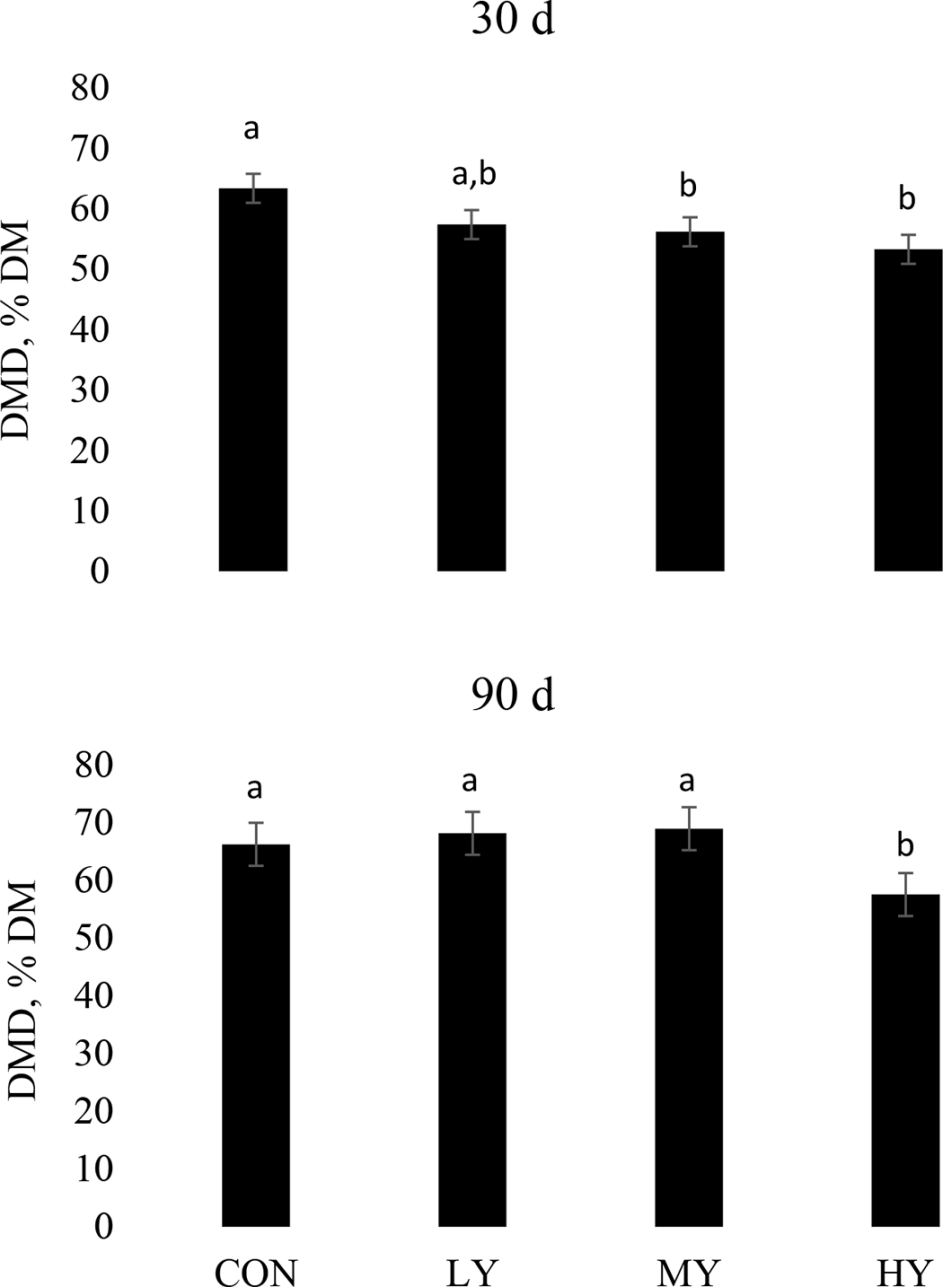
The 7-h in vitro degradability of DM (DMD; % DM) from rehydrated corn grain silage (RCGS) stored for 30 and 90 d and incubated with varying amounts of wild yeasts. CON = no addition of wild yeast; LY = addition of wild yeast to achieve projected counts of 5.3 log cfu/g of RCGS; MY = addition of wild yeast to achieve projected counts of 6.3 log cfu/g of RCGS; HY = addition of wild yeast to achieve projected counts of 7.3 log cfu/g of RCGS. Bars with different letters differ (*P* < 0.05). SEM = 2.40 and 3.72 for 30 and 90 d, respectively. Errors bars are the greatest SEM.

Although the current study assessed the effects of wild yeast inoculation on RCGS, it is important to acknowledge that this approach does not fully indicate the complexity of interactions in the field (i.e., molds and undesirable bacteria). In addition, our study did not evaluate a single strain of yeast and results should be considered with caution. Therefore, further research is warranted to elucidate the complexity of microbial dynamics and to characterize the metabolic activity of yeasts by isolating single strains. Overall, this study provides additional information regarding the nutrient composition, fermentation profile, and aerobic stability of RCGS with wild yeast inoculation. At 30 d, silage had greater yeast counts and a less pronounced fermentation. Contrary to our hypothesis, wild yeast inoculation in RCGS did not largely influence silage nutritive value, except for degradability of DM, and had minimal effects on RCGS at 90 d of storage. Moreover, high wild yeast populations can reduce in vitro DM degradability, which could possibly affect performance of dairy cows. Further research is warranted to identify wild yeast populations as their presence is dependent on their species and metabolism (Pahlow et al., 2003).
